# ﻿Water beetles (Coleoptera) associated with Afrotemperate Forest patches in the Garden Route National Park, South Africa

**DOI:** 10.3897/zookeys.1182.102866

**Published:** 2023-10-19

**Authors:** Matthew S. Bird, David T. Bilton, Musa C. Mlambo, Renzo Perissinotto

**Affiliations:** 1 Department of Zoology, University of Johannesburg, Auckland Park 2006, Johannesburg, South Africa University of Johannesburg Johannesburg South Africa; 2 Marine Biology and Ecology Research Centre, School of Marine Science and Engineering, Plymouth University, Drake Circus, Plymouth PL4 8AA, UK Plymouth University Plymouth United Kingdom; 3 Department of Freshwater Invertebrates, Albany Museum, Department of Zoology and Entomology, Rhodes University, Makhanda, 6139 Grahamstown, South Africa Rhodes University Grahamstown South Africa; 4 Coastal and Marine Research (CMR), Nelson Mandela University, P.O. Box 77000, 6031 Gqeberha, South Africa Nelson Mandela University Gqeberha South Africa

**Keywords:** aquatic Coleoptera, aquatic invertebrates, biodiversity census, forest conservation, freshwater biodiversity, southern Cape, temperate forests

## Abstract

Southern Afrotemperate Forest is concentrated in the southern Cape region of South Africa and whilst it is relatively well known botanically, the fauna, specifically the aquatic invertebrate fauna, is poorly documented. The majority of remaining intact forest habitat is contained within the Garden Route National Park (GRNP), which straddles the provincial boundary between the Western and Eastern Cape. This study undertakes a survey of the water beetle fauna inhabiting the GRNP. The aquatic ecosystems within temperate forests of the region are poorly researched from an ecological and biodiversity perspective, despite being known to harbour endemic invertebrate elements. We collected water beetles and in situ physico-chemical data from a total of 31 waterbodies across the park over two seasons (summer and late winter) in 2017. The waterbodies sampled were mostly small freshwater perennial streams and isolated forest ponds. A total of 61 beetle taxa was recorded (29 Adephaga, 32 Polyphaga) from these waterbodies. The water beetle fauna of these forests appears to be diverse and contains many species endemic to the fynbos-dominated Cape Floristic Region, but very few of the species appear to be forest specialists. This is in contrast to the fynbos heathland habitat of the region, which harbours a high number of water beetle species endemic to this habitat, often with Gondwanan affinity. Our study is the first to document the water beetles of Afrotemperate Forests in the southern Cape region and provides an important baseline for future work on such habitats in the region and in other parts of southern Africa.

## ﻿Introduction

Closed-canopy evergreen indigenous forest is a relatively scarce biome in southern Africa, most of this vegetation type in South Africa being located in the east and north of the country (Mucina and Geldenhuys 2006). Remaining forests in southern Africa are highly fragmented, existing as a series of ecological islands within a mosaic of other biomes, including savannah, grassland, fynbos, alien vegetation and agriculturally transformed lands. Additionally, the majority of remaining forests are small (< 100 ha), the distribution of patches showing what often appears to be a relictual pattern, for example, as fire refugia within other biomes, suggesting that forest fragmentation has been driven substantially by non-anthropogenic factors, particularly the development of fire prone ecosystems since the Miocene-Pliocene (e.g., Eeley et al. 1999). The largest single forest in South Africa is in the vicinity of Knysna in the southern Cape (25,706 ha), itself sitting within a much larger complex of forest patches totalling over 60,000 ha (Mucina and Geldenhuys 2006). The forests in the southern and western Cape regions have floristic affinities with Afromontane Forest in the Drakensberg and on mountains further north into tropical Africa, the southerly latitude allowing these forests to occur at relatively low altitude (Meadows and Linder 1989). These Cape forests are compositionally distinct from those further east and north, however, and usually considered to comprise a separate vegetation unit, Southern Afrotemperate Forest (Mucina and Geldenhuys 2006). Whilst some patches of this habitat occur in fire refugia, such as ravines and mountainsides across the Western Cape Province (and indeed into the Northern Cape), the majority is found in the coastal hills and low mountains that straddle the borders of the Western and Eastern Cape provinces. Whilst most of the historical extent of this forest has been lost to human activities (Geldenhuys 1991), more than 50% of what remains is protected within the Garden Route National Park (Mucina and Geldenhuys 2006).

Our understanding of the biodiversity of Afrotemperate Forests in southern Africa remains limited and patchy, both taxonomically and geographically. Floristic composition and endemism are relatively well understood for most groups; Southern Afrotemperate forests being dominated by palaeoendemic trees such as the podocarps (*Afrocarpus* and *Podocarpus*) and *Cunonia*, *Ocotea*, and *Olea*, but with relatively few strictly endemic plant taxa (Mucina and Geldenhuys 2006). A number of forest birds, including the Knysna Turaco (also known locally as the Knysna Lourie) *Tauracocorythaix* (Wagler, 1827) are near-endemic to the region, and amongst other vertebrates these forests support the near-endemic shrew *Myosorexlongicaudatus* Meester & Dippenaar, 1978 (Hilton-Taylor 2000), some near-endemic amphibians (Passmore and Carruthers 1995) and the strictly endemic Knysna Dwarf Chamaleon *Bradypodiondamarnum* (Boulenger, 1887) (Tolley et al. 2004; Stuart-Fox and Moussalli 2007). The invertebrate fauna of the southern Cape forests is much more poorly documented, but is known to include a number of apparent endemics (for a review of the soil fauna of South African forests, see Janion-Scheepers et al. 2016), in groups as diverse as land snails (e.g., Moussalli et al. 2009; Perera et al. 2021), millipedes (Hamer 1998), terrestrial isopods (Ferrara and Taiti 1979) and terrestrial beetles (e.g., Solidovnikov and Newton 2004; Janák and Makranczy 2016).

Very little work has been conducted on the freshwaters of Southern Afrotemperate Forests to date. Midgley and Schafer (1992) examined correlates of water colour in southern Cape streams, establishing that black water streams could occur in both fynbos and forested catchments. De Moor and Bellingham (2019) document and discuss the Trichoptera of streams in the region, including forested sites, and highlight the presence of a number of range-restricted Cape Floristic Region (CFR) endemic species. Otherwise, knowledge of the insect fauna of these habitats is largely limited to descriptions in the taxonomic literature (e.g., Solidovnikov and Newton 2004; Perkins 2009; Janák and Makranczy 2016). In South Africa, comparatively less is known about aquatic beetles (but see Perissinotto et al. 2016; Bird et al. 2017) unlike their counterparts in groups such as dung beetles (Davis et al. 2020), canopy beetles (Swart et al. 2021) and fruit chafers (Beinhundner 2017). Beetles have colonised water many times from separate terrestrial ancestors, forming an ecological grouping rather than a clade (Bilton et al. 2019). As a consequence, aquatic beetles are ecologically and functionally diverse and occupy the entire spectrum of freshwater habitats, where they often make up a significant proportion of freshwater macroinvertebrate biodiversity (Jäch and Balke 2008), making them an excellent group for ecological assessment (e.g., Bilton et al. 2006; Sánchez-Fernández et al. 2006; Picazo et al. 2011). The present study aimed to document the water beetle fauna of forested regions of the Garden Route National Park, through targeted surveys. Here we document all water beetle species recorded, together with multivariate analyses of water beetle assemblage composition. Given the general lack of such data from southern Africa (Bird et al. 2019), and Cape forests in particular (de Moor and Day 2013), our study provides a valuable baseline for the study of this key group of freshwater macroinvertebrates in Afrotemperate Forest habitats in southern Africa.

## ﻿Materials and methods

### ﻿Study area

**Figure 1.**
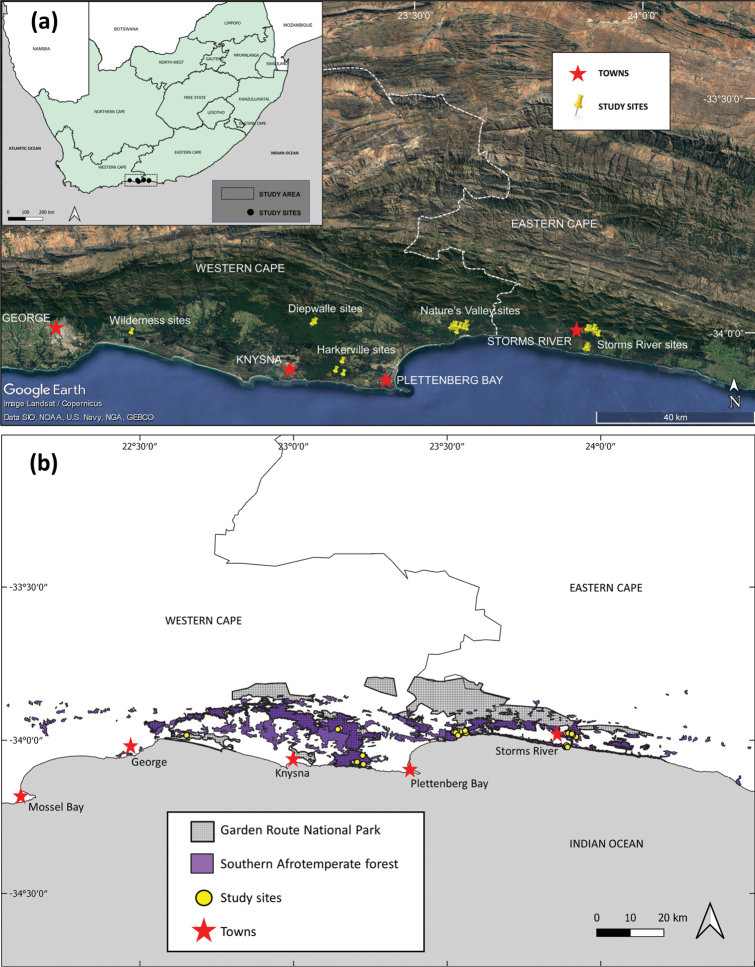
Location of the study sites along the southern Cape coastline of South Africa. Sites were grouped according to five main areas that represent different forest fragments within the Garden Route National Park (GRNP): Storms River; Nature’s Valley; Harkerville; Diepwalle and Wilderness (**a**). The position of the study sites in relation to the remaining core Afrotemperate Forest habitat in the region is depicted, as well the boundaries of the GRNP (**b**).

Samples were collected from a total of 31 waterbodies spanning the length of the Garden Route National Park (GRNP) along the southern Cape coast of South Africa between the towns of Storms River in the east and George in the west (Fig. 1a, b). This region constitutes the core area of remaining Southern Afrotemperate Forest habitat (sensu Mucina and Geldenhuys 2006), with only small, scattered fragments of this habitat occurring outside of the study area (Fig. 1b). The study area spans the border of the Western Cape and Eastern Cape provinces (Fig. 1a, b) and is part of a relatively moist coastal plain (the Mean Annual Precipitation, MAP, for Southern Afrotemperate Forest is 862 mm; Mucina and Geldenhuys 2006), which gives way to the semi-desert Karoo inland, as apparent in the satellite imagery of Fig. 1a. Despite covering a relatively small area, Southern Afrotemperate Forest habitat is considered ‘Least Threatened’ (Mucina and Geldenhuys 2006) thanks largely to the statutory protection it receives, with more than half of the extent of these forests falling within the boundaries of the GRNP (Fig. 1b).

**Figure 2.**
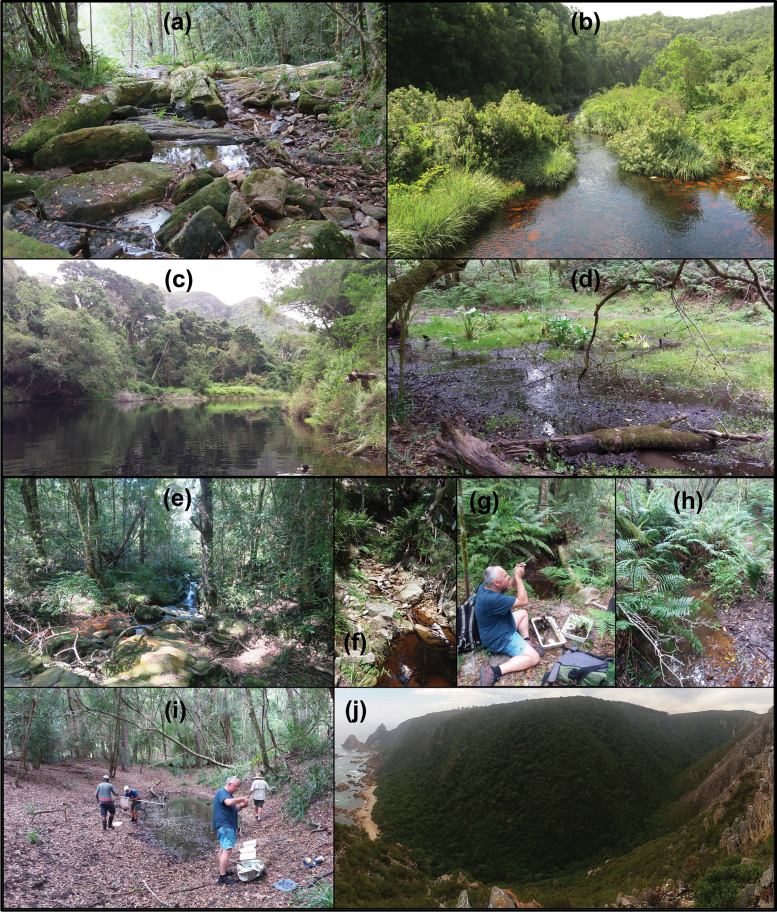
Examples of the waterbodies and habitat types sampled in the Garden Route National Park (pictures taken during the summer survey in mid-February 2017). **a** Stream in the Plaatbos forest, Storms River (site 4) **b** main channel of the Storms River where the bridge crosses near the public picnic site (site 6) **c** the main channel of the Groot River at Nature’s Valley (site 13) **d** marshy pond at Plaatbos, Storms River (site 3) **e** stream crossing a hiking trail in the Plaatbos forest, Storms River (site 2) **f** small stream on the Kalanderkloof hiking trail, Nature’s Valley (site 9) **g** DTB examining water beetles at a pond in the Harkerville forest (site 15) **h** pond in the Diepwalle forest (site 19) **i** the authors hard at work sampling a pond at Nature’s Valley (site 12) adjacent to the Groot River **j** typical Southern Afrotemperate Forest habitat at Harkerville.

Sample collection was performed across two seasons, with 20 sites being sampled in early February 2017 (mid-summer, hereafter ‘summer’) and 14 sites sampled in mid-September 2017 (late winter, hereafter ‘winter’). Three of the sites sampled in summer were sampled again in winter. In terms of waterbody types sampled in this study, 15 of the sites sampled in summer were small perennial streams, whilst four such sites were sampled in winter. Five sites sampled in summer were ponds, whilst nine ponds were sampled in winter. One of the sites sampled in winter was a seepage wetland. The waterbodies were low lying, all occurring at less than 400 m altitude. Several clusters of sites were sampled, with 13 sites occurring in the vicinity of Storms River, 12 sites at Nature’s Valley, three at Harkerville, two at Diepwalle and one site was sampled at Wilderness. All sampled sites occurred within patches of Southern Afrotemperate Forest and were in a relatively pristine condition, being located inside the GRNP. The site locality information for all sampled waterbodies is provided in Table 1 and photographs of typical habitats are provided in Fig. 2.

**Table 1.** Site locality information for waterbodies sampled during this study. Two collection trips were undertaken, the first being during February 2017 (mid-summer) and the second during September 2017 (late winter). SR: Storms River; NV: Nature’s Valley; HV: Harkerville; DW: Diepwalle; WN: Wilderness.

Site	Date sampled (dd/mm/yyyy)	GPS (DD)	Altitude (m)	Region	Waterbody type	February (summer)	September (winter)
1	07/02/2017	-34.02138, 23.88472	41	SR	Stream	X	
2	07/02/2017; 14/09/2017	-33.97711, 23.89476	237	SR	Stream	X	X
3	07/02/2017; 14/09/2017	-33.97541, 23.90689	239	SR	Pond	X	X
4	07/02/2017	-33.98300, 23.90829	195	SR	Stream	X	
5	07/02/2017	-33.98180, 23.91132	233	SR	Stream	X	
6	07/02/2017	-33.98871, 23.91929	78	SR	Stream	X	
7	08/02/2017	-33.97638, 23.88886	228	SR	Stream	X	
8	08/02/2017	-33.97800, 23.88846	224	SR	Stream	X	
9	09/02/2017	-33.97403, 23.55288	64	NV	Stream	X	
10	09/02/2017	-33.97158, 23.54332	137	NV	Stream	X	
11	09/02/2017	-33.96859, 23.55978	3	NV	Stream	X	
12	09/02/2017; 15/09/2017	-33.96860, 23.55861	9	NV	Pond	X	X
13	09/02/2017	-33.97605, 23.56169	2	NV	Pond	X	
14	10/02/2017	-33.97428, 23.51926	39	NV	Stream	X	
15	11/02/2017	-34.05024, 23.22491	240	HV	Pond	X	
16	11/02/2017	-34.07092, 23.20679	189	HV	Stream	X	
17	11/02/2017	-34.07839, 23.22742	177	HV	Stream	X	
18	11/02/2017	-33.96131, 23.15123	392	DW	Stream	X	
19	11/02/2017	-33.96436, 23.14399	381	DW	Pond	X	
20	12/02/2017	-33.98355, 22.65148	5	WN	Stream	X	
21	14/09/2017	-34.02140, 23.8886	25	SR	Stream		X
22	15/09/2017	-34.01701, 23.88892	101	SR	Stream		X
23	15/09/2017	-34.02209, 23.89196	68	SR	Pond		X
24	15/09/2017	-33.98311, 23.90889	195	SR	Seep		X
25	15/09/2017	-33.97967, 23.90582	217	SR	Stream		X
26	15/09/2017	-33.96713, 23.56006	3	NV	Pond		X
27	16/09/2017	-33.96937, 23.53168	218	NV	Pond		X
28	16/09/2017	-33.96966, 23.52587	223	NV	Pond		X
29	16/09/2017	-33.97414, 23.52207	36	NV	Pond		X
30	16/09/2017	-33.97509, 23.52778	87	NV	Pond		X
31	16/09/2017	-33.98408, 23.53546	4	NV	Pond		X

### ﻿Field sampling protocol and beetle identification

Water beetles were collected during both seasons using sweep netting. A long-handled square-framed pond net with a 30-cm mouth and 1-mm mesh was used for this purpose. With each sweep the net would be swept from the water surface to the bottom substrate and back to the surface again, in similar fashion to the protocols of Perissinotto et al. (2016) and Bird et al. (2017). Submerged fringing vegetation and the shore margins were targeted, given the authors’ previous experience of finding most water beetles in these habitats. Visual searching of the margins of each waterbody for shore beetles and semi-aquatic taxa was conducted in addition to sweep netting. Sampling was continued until no additional new taxa were found, each location typically being worked by the team for ca. 1 h. All beetle specimens were killed using ethyl acetate vapour and preserved in absolute ethanol.

To provide baseline information on the freshwater habitats of GRNP, and an environmental context for the water beetle assemblages, basic in situ physico-chemical parameters were measured at each site. Temperature, conductivity, pH, turbidity, and dissolved oxygen were recorded using a YSI 6600-V2 multi-system probe. Physico-chemical measurements could not be taken from two of the sites during the summer survey due to logistical constraints.

All identifications were conducted by DTB, using a wide range of literature and, in some cases, comparison with reference/voucher material. All identifications were based, at least in part, on the study of male genitalia, unless otherwise stated.

### ﻿Data analysis

Spatio-temporal patterns in the physico-chemistry of the waterbodies were assessed to determine whether the beetle assemblages mirrored physico-chemical patterns. Differences in physico-chemistry amongst sampled waterbodies were depicted using Principal Components Analysis (PCA), after first normalising the variables in the matrix. The variables constituting each matrix were temperature, conductivity, dissolved oxygen, pH, depth, and turbidity. Physico-chemical differences were compared across three factors of interest, which were overlaid on the PCA plots: season (summer, winter); region (Storms River, Nature’s Valley, Harkerville, Diepwalle, Wilderness); and waterbody type (streams, ponds, seeps). Permutational MANOVA (PERMANOVA, Anderson 2001) was used to test for differences in waterbody physico-chemistry across each of the three factors above (i.e., between seasons, regions, and waterbody types). For the regional comparison, ‘Wilderness’ was excluded as a factor as no physico-chemical data were collected at the Wilderness site. For comparison of waterbody types, ‘seeps’ was excluded as a factor because only one seep was sampled on one occasion (i.e., streams were compared with ponds).

Spatio-temporal patterns in beetle assemblage composition were depicted using non-metric multidimensional scaling (MDS). The MDS plots were overlaid by the same factors as per the physico-chemical data (seasons, regions, and waterbody types). Beetle presence-absence data were converted to a Bray-Curtis dissimilarity matrix in order to construct the MDS plots. PERMANOVA was used to test for differences in beetle assemblage composition (represented by a Bray-Curtis dissimilarity matrix) between the two seasons sampled (February 2017 – mid-summer vs. September 2017 – late winter) and amongst the different regions (i.e., separate forest patches) sampled along the Tsitsikamma coast (Storms River, Nature’s Valley, Harkerville, Diepwalle), as well as between waterbody types (streams vs. ponds). For regional comparisons using PERMANOVA, Wilderness was not included in the test due to only one site being sampled in that region, and similarly seeps were excluded from the comparisons of waterbody types due to only one seep being sampled. Species richness (number of species recorded per waterbody) was similarly compared amongst seasons, regions, and waterbody types. Richness patterns were visually assessed using boxplots and comparisons between seasons, regions and waterbody types were performed using t-tests (two-group comparisons) or one-way ANOVA (three-group comparisons), given that the richness data followed a Gaussian distribution and significant heteroscedasticity was not evident for any of the comparisons (Quinn and Keough 2002). Lastly, beetle assemblage composition was regressed against the various environmental variables recorded in this study to determine what variables best account for the assemblage distribution patterns in the GRNP. The predictor variables here were the spatio-temporal variables (latitude, longitude, altitude, season), regional variables (Storms River, Nature’s Valley, Harkerville, Diepwalle), waterbody type (stream, pond, seep) and physico-chemistry (temperature, conductivity, dissolved oxygen, pH, depth, turbidity). Once again, Wilderness was not included as a regional factor (no physico-chemical data for this site). Multivariate regressions were performed using distance-based Redundancy Analysis (dbRDA, Legendre and Anderson 1999; McArdle and Anderson 2001). Separate marginal tests were first run between each environmental variable and beetle assemblage composition, followed by a step-wise selection of the environmental variables using the adjusted Akaike Information Criterion (AICc), which is recommended for small sample sizes (Burnham and Anderson 2002). A ‘best’ (most parsimonious) overall model was also calculated by considering all variable subsets, with parsimony scored according to the AICc criterion.

All tests were performed using an a priori significance level of α = 0.05. P values for PERMANOVA models were tested using 999 unrestricted permutations of the raw data. The PCA, MDS, PERMANOVA and DISTLM procedures were implemented with PRIMER v. 7.0.21 software (Clarke and Gorley 2015) with the PERMANOVA+ add-on (Anderson et al. 2008). Boxplots and univariate tests were performed using GraphPad Prism v. 9.1.0 for Windows (GraphPad Software, San Diego, California USA).

## ﻿Results

### ﻿Physico-chemical characteristics of the waterbodies

The waterbodies encountered in the forests of the GRNP were predominantly small perennial rocky streams, although a small proportion of these streams (e.g., sites 9 and 10) are expected to dry up intermittently. There are several larger running waters in the park, such as the Groot, Storms, and Salt rivers, which were also sampled in this study. The second-most abundant waterbody type encountered was ponds (or depression wetlands according to the South African wetland classification system; Ollis et al. 2013), which were all small and shallow and likely dry up on occasion.

Table 2 presents a summary of the physico-chemical variables recorded during each of the two surveys. Surface water temperatures appeared to be somewhat moderated by the shady forest and relatively mild coastal climate in this region, and water temperatures never exceeded 21.5 °C during the mid-summer survey, nor were the minimum winter temperatures extreme, never dropping below 12 °C. Differences in water temperature between summer and winter were not particularly pronounced, with the difference in median temperature of the waterbodies between the two seasons being approximately 5 °C. All the sites sampled were fresh (median summer and winter conductivity was 0.281 mS.cm^-1^ and 0.412 mS.cm^-1^ respectively), with only site 26 being slightly brackish (conductivity of 4.605 mS.cm^-1^). Median pH was circum-neutral in summer (6.76) and no sites were notably alkaline, however five of the sites were genuinely acidic (pH < 6). In winter, the sites visited were neutral-to-alkaline, with seven genuinely alkaline sites (pH > 8) and median pH was alkaline (8.06). Interestingly, sites 2 and 3, which were revisited in the winter survey, showed a substantial shift in their pH from acidic to alkaline conditions from summer to winter (Table 2).

**Table 2.** Physico-chemical variables recorded at each waterbody during the February and September 2017 surveys. Median, minimum, and maximum values are reported for each survey. Readings were not recorded at sites 13 and 20.

Survey date	Site	Temperature (°C)	Conductivity (mS.cm^-1^)	pH	Dissolved O_2_ (mg.L^-1^)	Turbidity (NTU)	Depth (m)
February 2017	1	20.41	0.980	7.80	8.45	1.6	0.10
	2	18.54	0.166	4.64	8.19	0.5	0.16
	3	20.41	0.221	6.41	2.53	8.0	0.17
	4	18.71	0.175	5.65	7.63	0.0	0.17
	5	18.64	0.163	5.31	2.35	1.4	0.21
	6	20.61	0.100	4.49	8.76	1.8	0.17
	7	19.95	1.138	6.74	7.53	1.8	0.10
	8	18.51	0.350	7.07	3.48	0.7	0.09
	9	17.51	0.811	6.91	6.22	0.2	0.16
	10	20.38	0.425	7.01	5.68	9.6	0.12
	11	21.44	0.178	6.89	3.83	13.2	0.11
	12	19.57	0.757	7.02	5.90	52.5	0.23
	14	20.93	0.250	5.51	9.09	210.0	0.07
	15	18.90	0.240	6.70	0.56	12.0	0.22
	16	18.50	0.373	6.87	8.82	0.0	0.60
	17	18.71	0.415	6.78	7.56	0.8	0.18
	18	17.52	0.193	6.35	3.31	11.6	0.30
	19	19.38	0.311	6.81	0.72	86.5	0.45
	**Median**	**19.14**	**0.281**	**6.76**	**6.06**	**1.8**	**0.17**
	**Minimum**	**17.51**	**0.100**	**4.49**	**0.56**	**0.0**	**0.07**
	**Maximum**	**21.44**	**1.138**	**7.80**	**9.09**	**210.0**	**0.60**
September 2017	2	13.48	0.955	8.16	9.10	2.1	0.30
	3	12.08	0.140	8.53	1.82	7.1	0.07
	12	14.93	0.802	7.32	1.53	12.9	0.05
	21	13.92	0.761	9.70	9.75	2.2	0.45
	22	13.74	1.724	8.18	2.46	28.6	0.60
	23	14.52	2.245	7.70	0.83	431.0	0.50
	24	12.72	0.454	8.15	3.41	12.5	0.05
	25	14.24	0.138	7.15	7.83	5.1	0.05
	26	14.54	4.605	7.78	5.93	26.2	0.05
	27	14.20	0.159	7.64	7.71	20.1	0.15
	28	15.28	0.334	6.70	6.51	5.2	0.07
	29	15.00	0.359	9.65	3.17	28.4	0.15
	30	17.54	0.279	8.77	6.88	31.5	0.08
	31	15.63	0.370	7.98	4.88	107.0	0.07
	**Median**	**14.37**	**0.412**	**8.06**	**5.41**	**16.5**	**0.08**
	**Minimum**	**12.08**	**0.138**	**6.70**	**0.83**	**2.1**	**0.05**
	**Maximum**	**17.54**	**4.605**	**9.70**	**9.75**	**431.0**	**0.60**

Sites were generally shallow, being < 1 m in depth (the deepest recording was 0.60 m for sites 16 and 22). However, this does not reflect the true depth of some of the larger rivers such as the Groot River, where water beetles were targeted in the shallow marginal vegetation at the edges (e.g., site 13) rather than the deeper middle section of the channel. Although some of the waterbodies were well oxygenated (dissolved oxygen concentrations > 7 mg.L^-1^), a large proportion of the sites had low dissolved oxygen concentrations, with some of the streams and ponds recording remarkably low values (< 2 mg.L^-1^, see Table 2). Median dissolved oxygen concentrations were low in both seasons (6.06 and 5.41 mg.L^-1^ for summer and winter respectively). Waterbodies were generally clear, as reflected by the relatively low median turbidity values (< 20 NTU in both seasons), although there were a few high turbidity exceptions (e.g., sites 14, 19, 23 and 31, see Table 2).

**Figure 3.**
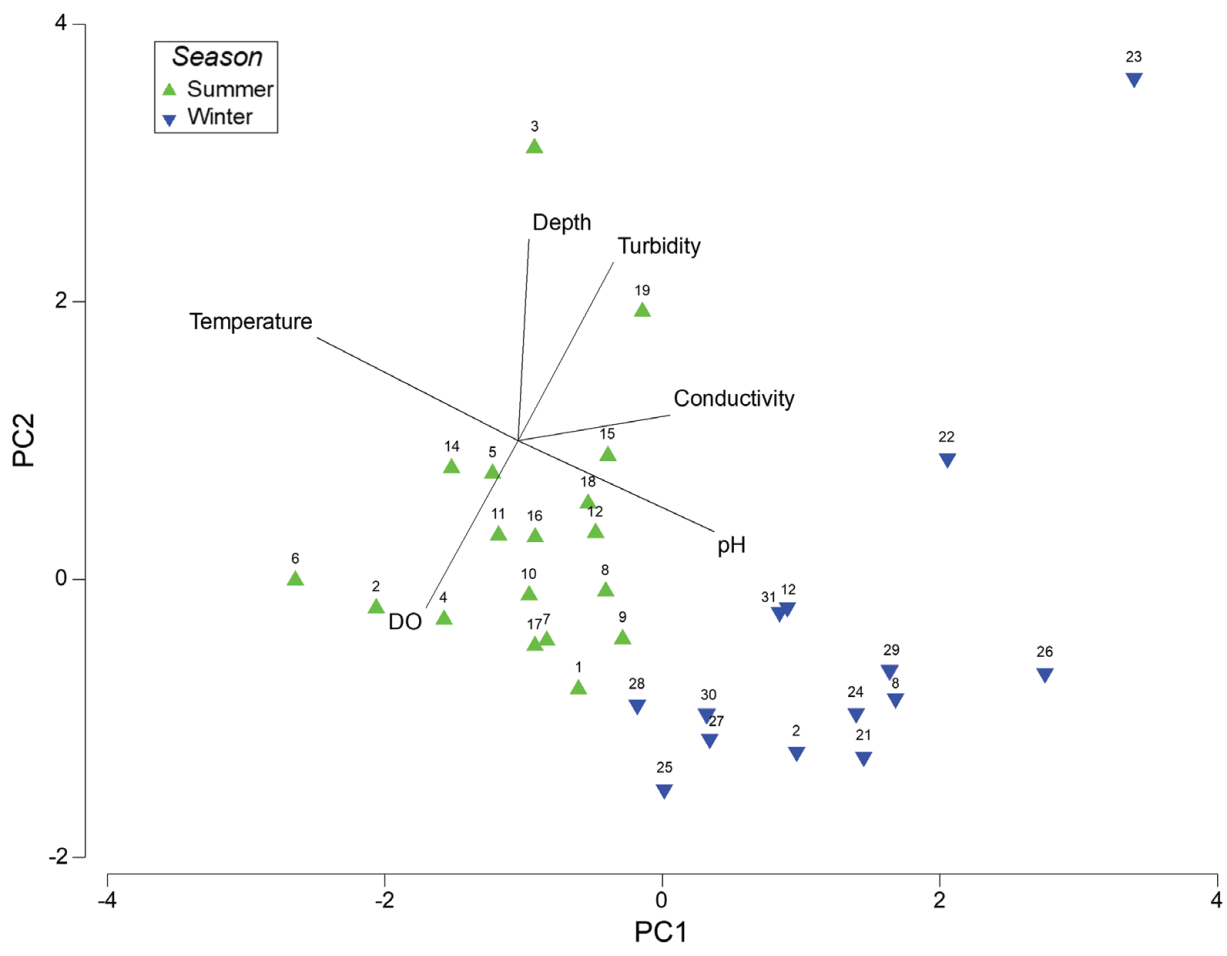
Principal components analysis depicting multivariate differences in the physico-chemistry of the various waterbodies sampled in this study. The site numbers are indicated above the symbol for each site and symbols have been differentiated according to season (summer vs. winter trips). The physico-chemical variables measured at each site have been overlaid as vectors on the plot.

According to the PCA plot in Fig. 3, waterbodies varied substantially in their overall physico-chemistry, but consistent gradients for each of the measured variables among the sites were not clear and the overlaid vectors were not well correlated with the sites on the plot. The only possible exceptions are for pH and temperature, with the winter sites towards the bottom right of the plot being associated with higher pH and lower water temperatures, the latter not being surprising. There was a clear distinction between the overall physico-chemical composition of waterbodies sampled in summer vs. winter, as evidenced by their separation on the plot (green vs. blue triangles). This separation was confirmed by the significant PERMANOVA test result for the factor ‘season’ (Table 3 (a)). No significant distinction in physico-chemistry was found among the waterbodies from the different regions of the park (Table 3 (b)), however physico-chemistry did differ between streams and ponds (Table 3 (c)).

**Table 3.** Non-parametric permutational MANOVA (PERMANOVA) results for models comparing the physico-chemistry of the waterbodies between (a) seasons, (b) regions and (c) waterbody types. The multivariate models tested for differences between group centroids in multivariate space, represented by Euclidean distance. An asterisk indicates significant P values at α = 0.05.

(a)	df	SS	MS	F	P
**Season**	1	45.64	45.64	9.75	0.001*
Residual	30	140.35	4.67	–	–
Total	31	186	–	–	–
**(b)**	**df**	**SS**	**MS**	**F**	**P**
**Region**	3	12.85	4.28	0.69	0.762
Residual	28	173.15	6.18	–	–
Total	31	186		–	–
**(c)**	**df**	**SS**	**MS**	**F**	**P**
**Waterbody type**	1	18.344	18.34	3.26	0.005*
Residual	29	163.04	5.62	–	–
Total	30	181.39	–	–	–

### ﻿Water beetles

The list of water beetles recorded in this study is reported in Table 4. In all 61 taxa were collected from the GRNP over the two surveys of this study, with 47 taxa recorded from the summer survey and 35 from the winter trip. Fifty-three taxa were identified to species level, whilst eight taxa were identified to genus (due to lack of modern revisions) and one to family (Ptilodactylidae larvae). Twenty-nine of the recorded taxa belong to the suborder Adephaga (predaceous water beetles) and 32 belong to the suborder Polyphaga. The richest family collected in this study was the Dytiscidae (Adephaga), with 22 species, followed by the Hydrophilidae (Polyphaga) with 14 taxa, and the Hydraenidae (Polyphaga) with nine species. Similarly, dytiscids were the most widespread family, occurring at 34 sites in the park, followed by the hydrophilids at 23 sites, and hydraenids at 14 sites. *Hydaticusgalla* Guérin-Méneville, 1849 (Dytiscidae) was the most widespread species in the GRNP, recorded from 23 waterbodies across the park, followed by *Copelatuscaffer* Balfour-Browne, 1939 (Dytiscidae) from 20 of the waterbodies, and *Copelatuscapensis* Sharp, 1882 (Dytiscidae) recorded from 17 sites. In contrast, 26 of the taxa were only recorded at a single waterbody. Thus, almost half of the taxa had a very localised distribution in this study.

**Table 4.** Water beetles collected from the Garden Route National Park during the course of this study. The sites are listed from which each taxon was collected on each of the two sampling trips (February and September 2017). Site numbers 1 – 31 correspond to those listed in Table 1. The regions where each taxon occurred are also indicated: SR – Storms River; NR – Nature’s Valley; HV – Harkerville; DW – Diepwalle; WN – Wilderness. + Taxa endemic to South Africa.

Taxa	Sampling date		Region				
	February	September	SR	NV	HV	DW	WN
Gyrinidae:							
*Dineutusgrossus* (Modeer, 1776)	1, 6, 8, 14	23	X	X			
+*Aulonogyrusformosusknysnanus* Brinck, 1955	13, 14, 16, 17			X	X		
*Aulonogyrusvarians* Brinck, 1955	6	25	X				
+*Orectogyruscapicola* Brinck, 1955	14			X			
Haliplidae:							
+*Haliplusexsecratus* Guignot, 1936	11, 20			X			X
Noteridae:							
*Synchortussimplex* Sharp, 1882	3		X				
Dytiscidae:							
+*Agabusaustellus* Englund, Bilton & Bergsten, 2020	15				X		
+*Copelatuscaffer* Balfour-Browne, 1939	1, 2, 3, 4, 10, 11, 12, 13, 15, 18, 19	2, 3, 12, 21, 23, 24, 25, 26, 30	X	X	X	X	
+*Copelatuscapensis* Sharp, 1882	1, 3, 9, 10, 11, 12, 13, 15, 18	3, 12, 21, 23, 24, 26, 28, 30	X	X	X	X	
*Copelatuserichsoni* Guérin-Méneville, 1849	10, 11, 12	3, 12, 23, 24, 30	X	X			
+*Copelatusnotius* Omer-Cooper, 1965		11		X			
*Aethionectesapicalis* (Boheman, 1848)	12	12		X			
*Hydaticuscapicola* Aubé, 1838	10, 11, 13, 14, 15, 17, 18, 20	12, 23, 27	X	X	X	X	X
*Hydaticusdregei* Aubé, 1838	8		X				
*Hydaticusgalla* Guérin-Méneville, 1849	1, 3, 4, 5, 7, 8, 10, 11, 12, 13, 14, 15, 16, 18, 19	3, 12, 21, 22, 23, 24, 27, 30	X	X	X	X	
+*Bidessusmundulus* Omer-Cooper, 1965		28		X			
*Clypeodytesmeridionalis* Régimbart, 1895	1, 3, 6, 8, 13,	25	X	X			
*Hydroglyphuslineolatus* (Boheman, 1848)		27		X			
*Uvarusopacus* (Gschwendtner, 1935)	3		X				
*Yolafrontalis* Régimbart, 1906	4, 6, 8, 11, 4		X	X			
+*Canthyporusfluviatilis* Omer-Cooper, 1956	3, 15		X		X		
+*Canthyporushottentottus* (Gemminger & Harold, 1868)	3, 8	26, 27	X	X			
+*Hydrovatusamplicornis* Régimbart, 1895	3	28	X	X			
+*Darwinhydrussolidus* Sharp, 1882	15	27, 28, 29, 31		X	X		
+*Hydropeplustrimaculatus* (Laporte, 1835)		27		X			
+*Hyphydrussoni* Biström, 1982	1, 3, 6, 7, 10, 11, 12, 13, 15, 16, 20	12, 22, 25, 26, 27	X	X	X		X
+*Africophilusjansei* Omer-Cooper & Omer-Cooper, 1957	14			X			
*Laccophiluslineatus* Aubé, 1838	3, 6, 7, 11, 12, 13, 14, 20	22, 25	X	X			X
Hydrochidae							
*Hydrochus* sp.		27, 28, 29		X			
Spercheidae							
*Spercheuscerisyi* Guérin-Méneville, 1842		27, 28		X			
Hydrophilidae							
*Amphiopsglobus* Erichson, 1843	1, 11, 12, 14, 19	12, 22	X	X		X	
*Amphiopssenegalensis* (Laporte, 1840)	13			X			
+*Anacaenacapensis* Hebauer, 1999		25	X				
+*Anacaenaglabriventris* Komarek, 2004	10, 14	27		X			
*Agraphydrusalbescens* (Régimbart, 1903)	6, 13		X	X			
+*Enochrushartmanni* Hebauer, 1998	27, 28, 29			X			
Enochrus (Methydrus) sp.	1, 3, 4, 8, 9, 10, 12, 13, 15, 17, 19	23, 24, 30	X	X	X	X	
*Helochareslongipalpis* (Murray, 1859)	3		X				
*Helochares* sp.	6		X				
+*Limnoxenussjoestedti* Knisch, 1924		27		X			
*Hydrocharaelliptica* (Fabricius, 1801)		27		X			
*Sternolophusmundus* (Boheman, 1851)	1, 11, 12, 13	12	X	X			
*Laccobiuspraecipuus* Kuwert, 1890	14			X			
*Coelostoma* sp.	14			X			
Hydraenidae							
*Hydraenacooperi* Balfour-Browne, 1954	3, 13		X	X			
+*Mesocerationapicalum* Perkins & Balfour-Browne, 1994	2, 4, 16, 17		X		X		
+*Mesocerationbarriotum* Perkins, 2008	17				X		
+*Mesocerationdissonum* Perkins & Balfour-Browne, 1994	2, 4, 5		X				
+*Mesocerationdistinctum* Perkins & Balfour-Browne, 1994	6		X				
+*Mesocerationintegrum* Perkins, 2008	17				X		
+*Nucleotopsinterceps* Perkins, 2004		29		X			
+*Parhydraenaasperita* Perkins, 2009	1, 2, 4, 15, 17	31	X	X	X		
+*Parhydraenaseriata* Balfour-Browne, 1954		22, 26, 29	X	X			
Dryopidae							
+*Strina* sp.	6, 17	25	X		X		
Elmidae							
*Stenelmis* sp.	2		X				
+*Elpidelmiscapensis* (Grouvelle, 1890)	2, 4, 6, 17	25	X		X		
+*Elpidelmisfossicollis* Delève, 1966		25	X				
+*Peloriolus* sp. 1	6		X				
+*Peloriolus* sp. 2	2, 5, 6, 17	25	X		X		
Ptilodactylidae							
Ptilodactylidae (larvae)	13			X			

**Figure 4.**
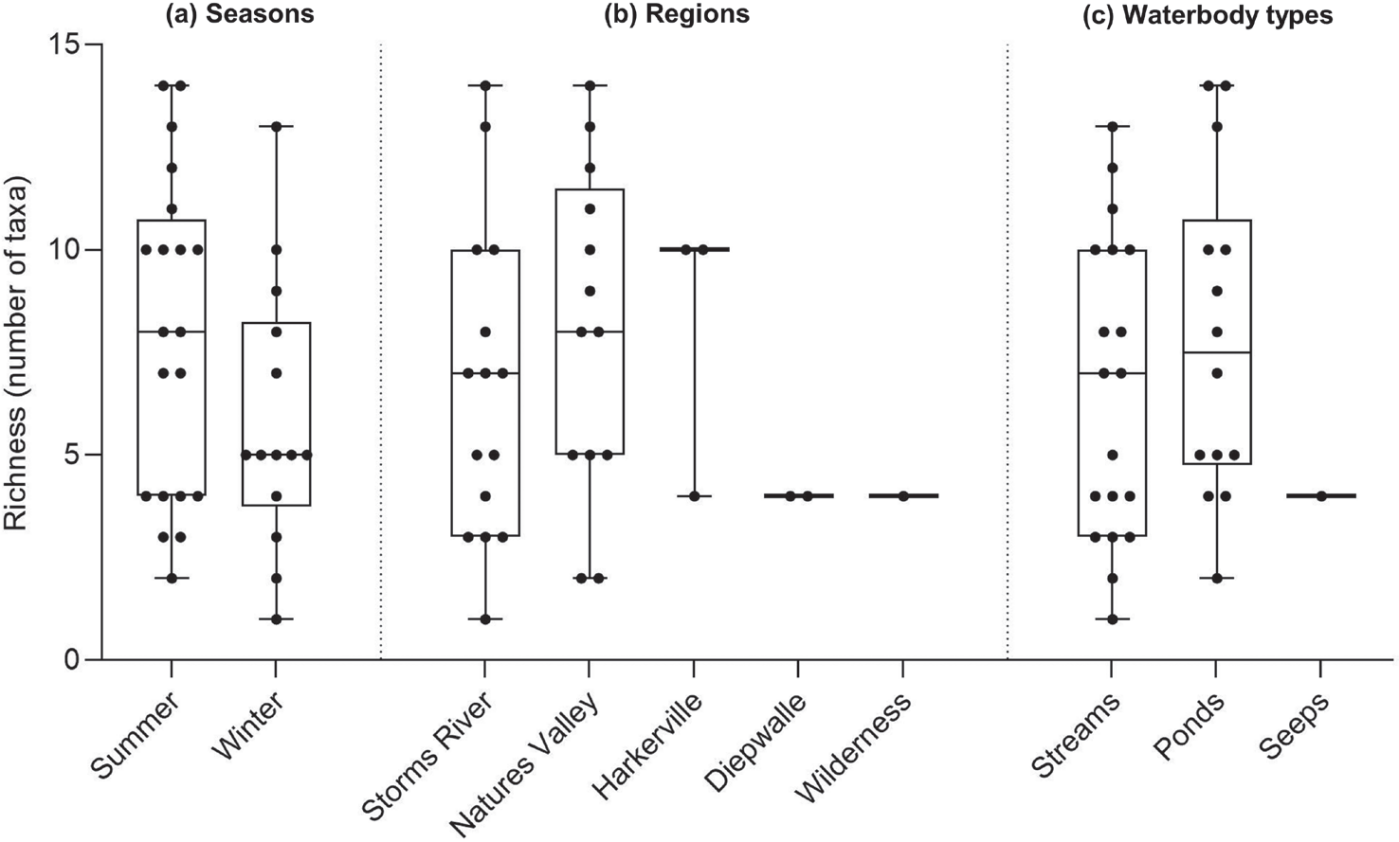
Boxplots comparing the median and spread of water beetle taxon richness (number of taxa per site) between **a** seasons **b** regions and **c** waterbody types at GRNP. The middle line represents the median, whilst the boxes demarcate the interquartile range and the ‘whiskers’ extend to the maximum and minimum values. The black circles on the graphs represent individual data points (number of taxa) for each site sampled. Unpaired t-tests reported no significant difference in richness between the two seasons (t_32_ = 1.604, p = 0.119) and between the waterbody types (streams vs. ponds, t_31_ = 0.959, p = 0.345). One-way ANOVA reported no significant difference in richness between the regions (F_3,29_ = 0.809, p = 0.499). ‘Seeps’ was excluded as a factor from the waterbody comparisons due to only one sample being taken from this habitat and ‘Wilderness’ was similarly excluded from the regional comparison due to only one sample being collected in this region.

Mean taxon richness across all sites and sampling trips was 7.1±3.7 (±SD) taxa per site. The most taxa recorded at a single site was 14, recorded at sites 3 and 13, which were both ponds. This was followed by sites 6 (stream) and 27 (pond), where 13 taxa were collected at each of these sites. Therefore, three out of the four richest sites were ponds. The boxplots in Fig. 4 indicate that the median taxon richness of water beetles was higher in summer than winter, but that there was no overall significant difference between the seasons (t_32_ = 1.604, p = 0.119). In terms of regions, Nature’s Valley sites had a higher median richness than for Storms River, but no overall significant difference in richness was reported across the regions (F_3,29_ = 0.809, p = 0.499). Ponds had slightly higher median richness than streams, but once again the difference was not significant (t_31_ = 0.959, p = 0.345).

**Figure 5.**
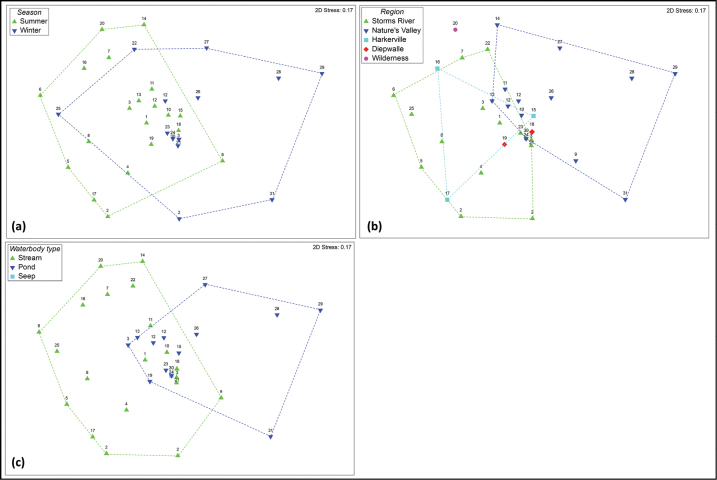
Multidimensional scaling (MDS) plots depicting the similarity of sites sampled at GRNP in terms of their water beetle assemblages. Symbols on the plot have been coded in terms of **a** season **b** region and **c** waterbody type. Convex hulls (dashed lines) have been overlaid on each plot to clarify groupings according to season, region, or waterbody type.

Water beetle assemblage composition differed between seasons, regions, and waterbody types at GRNP, as depicted visually in the MDS plots in Fig. 5. These differences were significant according to the PERMANOVA test results (Table 5). The summer and winter sites do show some overlap in Fig. 5a towards the middle of the plot, but the group centroids are significantly different. In terms of regions, the Nature’s Valley sites form a fairly distinct cluster towards the right of the plot (Fig. 5b), with Storms River, Harkerville, and Diepwalle sites mostly overlapping in their beetle assemblage composition (towards the left of the plot). As observed for seasons, the stream and pond waterbody types showed some overlap in their beetle faunas (towards the middle of the plot in Fig. 5c), but overall their group centroids were distinct.

**Table 5.** Non-parametric permutational MANOVA (PERMANOVA) results for models comparing beetle assemblage composition across (a) seasons, (b) regions and (c) waterbody types. The multivariate models tested for differences between group centroids in Bray-Curtis dissimilarity space. SR: Storms River; NV: Nature’s Valley; HV: Harkerville; DW: Diepwalle. For the regional comparison, Wilderness was not included due to only one site being sampled there on one occasion and for the comparison of waterbody types, ‘seeps’ was excluded as a factor because only one seep was sampled on one occasion (i.e., streams were compared with ponds). An asterisk indicates significant P values at α = 0.05.

**(a)**	**df**	**SS**	**MS**	**F**	**P**			
**Season**	1	6241.6	6241.6	2.20	0.018*			
Residual	32	90669	2833.4	–	–			
Total	33	96910	–	–	–			
**(b)**	**df**	**SS**	**MS**	**F**	**P**	***Post hoc pairwise comparisons***		
						**Groups**	**t**	**P**
**Region**	3	12497	4165.6	1.51	0.048*	SR, NV	1.477	0.021*
Residual	29	80134	2763.2	–	–	SR, HV	1.238	0.140
Total	32	92630	–	–	–	SR, DW	0.840	0.661
						NV, HV	1.265	0.103
						NV, DW	0.979	0.329
						HV, DW	1.282	0.155
**(c)**	**df**	**SS**	**MS**	**F**	**P**			
**Waterbody type**	1	6152.4	6152.4	2.136	0.033*			
Residual	31	89270	2879.7	–	–			
Total	32	95422	–	–	–			

The measured environmental variables in this study were together able to explain approximately 78.5% of the variation in beetle assemblage composition among the waterbodies sampled in the GRNP (Table 6 (a)). Although five of the variables were significantly associated with assemblage composition when considered independently (Table 6 (a)), only pH was selected in the step-wise (Table 6 (b)) and best overall (Table 6 (c)) AICc models when environmental variables were considered non-independently (i.e., accounting for the effects of other variables in the model). The most parsimonious model overall, considering all variable subsets, was that which included only pH. Despite being the most parsimonious, this model only accounts for ~8% of the variation in beetle assemblage composition and thus has very low explanatory power. Taken together, the results in Table 6 (a–c) indicate that, with the possible exception of pH, none of the individual environmental variables had a particularly strong influence on beetle assemblages, but cumulatively they were able to explain most (~ 78.5%) of the variation in assemblage composition between sites. This cumulative amount of explained variation is relatively high, considering that this study did not involve exhaustive sampling of all potential explanatory environmental variables.

**Table 6.** Results of the dbRDA multivariate regression tests of environmental variables against beetle assemblage composition. Independent marginal tests are first presented (a), followed by variables selected by the step-wise procedure using the AICc selection criterion (b) and the ‘best’ (most parsimonious, considering all combinations of variables) overall model according to the AICc criterion (c). ‘% Var’: the percentage of variation in each Bray-Curtis similarity matrix that is explained by the respective predictor variable in each test; ‘Cum. % var’: the cumulative percentage variation across all tests; ‘Res. df’: residual degrees of freedom associated with each test. An asterisk indicates significant variables at α = 0.05.

**(a) Marginal tests**:					
**Variable**	**F**	**P**	% **Var**		
Latitude	1.24	0.240	3.99		
Longitude	1.20	0.294	3.86		
Season	2.01	0.036*	6.31		
Altitude	1.05	0.405	3.40		
Region: ‘Storms River’	1.83	0.065	5.77		
Region: ‘Nature’s Valley’	2.31	0.019*	7.16		
Region: ‘Harkerville’	1.48	0.136	4.72		
Region: ‘Diepwalle’	0.70	0.691	2.29		
Waterbody type: ‘Stream’	2.21	0.021*	6.89		
Waterbody type: ‘Pond’	2.01	0.042*	6.29		
Waterbody type: ‘Seep’	0.45	0.842	1.49		
Temperature	1.85	0.053	5.84		
Conductivity	1.17	0.33	3.78		
DO	1.34	0.198	4.29		
pH	2.64	0.006*	8.11		
Depth	0.68	0.717	2.24		
Turbidity	0.66	0.702	2.18		
		Total:	78.59		
**(b) Sequential tests**:					
**Variable**	**AICc**	**F**	**P**	% **Var.**	**Res. df**
pH	256.1	2.64	0.01*	8.11	30
**(c) Best solution**:					
**Variable**	**AICc**	**F**	**P**	% **Var.**	**Res. df**
pH	256.1	2.64	0.01*	8.11	30

## ﻿Discussion

Our work demonstrates that the waterbodies of forests of the Garden Route National Park support a diverse water beetle fauna, including a number of South African endemics. The total of 61 taxa recorded from the region is, however, considerably lower than the 116 reported from similar surveys by the same team in the subtropical iSimangaliso Wetland Park, further north on the KwaZulu-Natal coast (Perissinotto et al. 2016; Bird et al. 2017). It is also lower than the typical diversity reported from tropical forest systems in Africa and elsewhere. For example, Bilardo and Rocchi (2011) recorded 51 species of aquatic Adephaga (vs. 29 in GRNP) in the Monts de Cristal National Park, Gabon. Apenborn (2013) reported the collection of 122 species of aquatic beetles, representing ten different families, in the Peruvian Amazon near the Panguana Biological Field Station (Hendrich et al. 2015). In northern temperate forests, water beetle biodiversity can also often be higher than we observed in the southern Cape. In Knyszyn Primeval Forest in north-east Poland, for example, Greń et al. (2022) reported 128 species of aquatic Coleoptera from this approximately 1,000 km^2^ site.

Of the species recorded here, 32 are endemic to South Africa. The vast majority of these are Cape endemics, more widespread in the fynbos biome to the west, and not tied to forest waterbodies. Such species include the dytiscids *Darwinhydrussolidus* Sharp, 1884 and *Hydropeplustrimaculatus* (Laporte, 1835), both of which are widespread and often abundant in lentic waters in fynbos in the far southwestern Cape, a number of the stream-dwelling *Mesoceration* (Hydraenidae) found in GRNP and the two lotic *Elpidelmis* species (Elmidae). Very few water beetle species found in these forests are either local endemics or forest specialists, the suite of taxa recorded during our surveys being dominated by species typical of fynbos waterbodies of the southern Cape (DTB, pers. obs.). Taxa which appear to be genuinely restricted to this region are *Aulonogyrusformosusknysnanus* Brinck, 1955 (Gyrinidae) and *Parhydraenaasperita* Perkins, 2009 (Hydraenidae). Of these two, only the latter appears to be predominantly a forest species, which is particularly abundant in the margins of small standing waters filled with decaying leaf litter, although it has also been reported from stream margins in the nearby Little Karoo (Perkins 2009). Otherwise, the only forest specialist discovered during our surveys is *Aethionectesapicalis* (Boheman, 1848), a relatively widespread, large Afrotropical diving beetle (Omer-Cooper 1966), typically breeding in fish-free temporary waters with dead leaves. Interestingly, this targeted survey did not reveal any species new to science, and forested waterbodies in the region appear to be genuinely almost devoid of narrow-range endemics. This finding is in stark contrast to the situation in fynbos-dominated catchments, particularly further west in the Cape, where recent work has revealed a large number of apparently locally endemic species, particularly in the Hydraenidae (e.g., Bilton 2013a, b, 2014a, b, 2015a, b, c; Bilton and Mlambo 2019). Recent sampling in other remnant patches of Southern Afrotemperate Forest in the Cape (e.g., Grootevanderbosch in the Langberg) have also failed to find any locally endemic water beetles and Southern Afrotemperate Forest streams in general appear to support fewer species and individuals of most water beetle groups than similarly sized systems in fynbos (DTB, pers. obs.). Possible reasons behind this pattern remain unclear, but may relate to palaeogeographic changes (e.g., Lewis 2008; Quick et al. 2016) and levels of autochthonous productivity, particularly biofilm composition and availability, which may be lower in small, heavily shaded catchments. In the case of vertebrates, Lawes et al. (2007) suggested that the relative paucity of local endemics in Southern Afrotemperate Forests has resulted through a combination of climatic extinction filtering during the Pleistocene and the infiltration of assemblages by generalist species from surrounding matrix habitats. The lack of forest-specialist water beetles in GRNP suggests that similar processes may apply to the aquatic insect faunas here.

Our study demonstrates that there are clear, measurable, differences between the aquatic beetle assemblages in different forested sections of the Garden Route National Park, as revealed by nMDS and PERMANOVA analyses, but no significant differences in species richness. Clearly, despite these forested catchments being close geographically, there is significant spatial variation in aquatic habitats, reflected in the different beetle faunas. Interestingly, the relatively few environmental parameters recorded during our study are able to explain almost 79% of the variation in beetle assemblage composition across sites, suggesting that these measures capture the main environmental drivers of species composition in the region. In most studies, even with many more environmental parameters, the proportion of explained variation is typically much lower (e.g., Rundle et al. 2002).

In summary, our study documents the aquatic beetle faunas of southern Cape Afrotemperate Forests for the first time, providing an important baseline for future work in the area and similar habitats in other parts of southern Africa. We show that these systems support a wide range of water beetle species, including a number of South African endemics, but do not, apparently, harbour any truly local endemics, even in running waters. This observation is in marked contrast to streams draining fynbos catchments, particularly further west in the Cape, where high concentrations of locally endemic water beetles are known, many with Gondwanan affinities. Whilst de Moor and Bellingham (2019) note that the Trichoptera of the region includes a number of Cape endemics, the degree to which these are locally endemic to the Garden Route remains unclear.

## ﻿Additional information

### Author ORCIDs

Matthew S. Bird https://orcid.org/0000-0001-9163-1008

David T. Bilton https://orcid.org/0000-0003-1136-0848

Musa C. Mlambo https://orcid.org/0000-0001-7624-5686

Renzo Perissinotto https://orcid.org/0000-0002-9224-3573

### Data availability

All of the data that support the findings of this study are available in the main text or Supplementary Information.
